# Recent Approaches for Downplaying Antibiotic Resistance: Molecular Mechanisms

**DOI:** 10.1155/2023/5250040

**Published:** 2023-01-23

**Authors:** Sarfraz Ahmed, Muhammad Zeeshan Ahmed, Safa Rafique, Seham Eid Almasoudi, Mohibullah Shah, Nur Asyilla Che Jalil, Suvash Chandra Ojha

**Affiliations:** ^1^Department of Basic Sciences, University of Veterinary and Animal Sciences Lahore, Narowal Campus, 51600 Narowal, Pakistan; ^2^Department of Biochemistry, Bahauddin Zakariya University, Multan 60800, Pakistan; ^3^School of Biochemistry and Biotechnology, University of the Punjab, 54590 Lahore, Pakistan; ^4^University of Tabuk, King Faisal Street, 47512 Tabuk, Saudi Arabia; ^5^Department of Pathology, School of Medical Sciences, Universiti Sains Malaysia, 16150 Kubang Kerian, Kelantan, Malaysia; ^6^Department of Infectious Diseases, The Affiliated Hospital of Southwest Medical University, 646000 Luzhou, China

## Abstract

Antimicrobial resistance (AMR) is a ubiquitous public health menace. AMR emergence causes complications in treating infections contributing to an upsurge in the mortality rate. The epidemic of AMR in sync with a high utilization rate of antimicrobial drugs signifies an alarming situation for the fleet recovery of both animals and humans. The emergence of resistant species calls for new treatments and therapeutics. Current records propose that health drug dependency, veterinary medicine, agricultural application, and vaccination reluctance are the primary etymology of AMR gene emergence and spread. Recently, several encouraging avenues have been presented to contest resistance, such as antivirulent therapy, passive immunization, antimicrobial peptides, vaccines, phage therapy, and botanical and liposomal nanoparticles. Most of these therapies are used as cutting-edge methodologies to downplay antibacterial drugs to subdue the resistance pressure, which is a featured motive of discussion in this review article. AMR can fade away through the potential use of current cutting-edge therapeutics, advancement in antimicrobial susceptibility testing, new diagnostic testing, prompt clinical response, and probing of new pharmacodynamic properties of antimicrobials. It also needs to promote future research on contemporary methods to maintain host homeostasis after infections caused by AMR. Referable to the microbial ability to break resistance, there is a great ultimatum for using not only appropriate and advanced antimicrobial drugs but also other neoteric diverse cutting-edge therapeutics.

## 1. Introduction

Antimicrobial drugs have provided solid support for clinical treatment since 1928, through the discovery of the first antibiotic in the form of penicillin. These have been proven to be effective in decreasing the mortality rate caused by bacterial infections such as pneumonia, tuberculosis (TB), and gastrointestinal [1]. The appropriate and inappropriate usage of antimicrobial drugs is increasing globally, reporting the emergence and spread of antimicrobial resistance (AMR) among diverse pathogens. AMR is expected to kill 10 million people by 2050 and cost the global economy $100 trillion [1]. It is hypothesized that microorganisms developed resistance to drugs over time through the process of Darwin's theory of natural selection of the fittest, adopting genetic variability and modifications [2]. Resistance of microbes has led to failures and stern complications in the treatment of bacterial infections, escalating morbidity and mortality around the world [3].

WHO and scientific literature have reported many microbes to be resistant to drugs, such as vancomycin-resistant *Enterococcus* (VRE), imipenem-resistant *Acinetobacter baumannii*, methicillin-resistant *Staphylococcus aureus* (MRSA), cephalosporin-resistant *Escherichia coli*, clarithromycin-resistant *Helicobacter pylori*, fluoroquinolone-resistant *Campylobacter* spp., fluoroquinolone-resistant *Salmonellae*, cephalosporin-resistant and fluoroquinolone-resistant *Neisseria gonorrhoeae*, penicillin-non-susceptible *Streptococcus pneumoniae*, ampicillin-resistant *Haemophilus influenzae*, fluoroquinolone-resistant *Shigella* spp., *Klebsiella*, *Serratia*, *Proteus*, and *Broccoli* [4]. These pathogens have become synonymously futile towards treatments and or therapeutic regimens, consequently resulting in a relentless public health concern [5]. The current microbial strains have not only gained resistance to a single medicine but also to numerous drugs with random and massive dissemination in the community [6]. It has also been reported that bacterial isolates have developed resistance against colistin and carbapenems which are assumed to be last-line stronghold antibiotics against Gram-negative bacteria (GNB), ultimately framing a serious threat [7].

Persistent reports of AMR pathogens have set up safety issues and have produced serious therapeutic challenges that may impend the global healthcare system [8]. These resistant bacteria are not limited to hospitals but are also transmitted through hospital-acquired infections (HAI), which represent not only grim health concerns but also an economic burden on a global level. Literature shows that HAIs affected >1.4 million people worldwide with a mortality rate of up to 10% [4]. Research has shown that AMR and its transmission are contributed through environmental factors like sanitation, waste management, drinking water, use of hygiene, and animal husbandry [9]. Agricultural fertilizers such as nitrogen fertilizers also disseminate AMR genes' emergence [10].

AMR is attributed to two main types of mechanisms. First is intrinsic resistance that is considered due to passive immunization, i.e., natural resistance and/or resistance through native genes, in which resistance against many antibiotics is supposed to be due to low membrane permeability and other genetic factors. Second is the adaptive resistance that is the result of evolutionary pressure, i.e., the bacterial class which was previously sensitive to the antimicrobial drugs now possesses a counterattack mechanism [11]. In bacterial strains, genes can be transferred from relatives or otherwise can be acquired from nonrelatives via mobile genetic carriers such as plasmids. This horizontal gene transfer (HGT) may lead to antibiotic resistance among diverse species of bacteria. Resistance can also pass off through spontaneous mutations [12]. Antibiotics compete and remove drug-sensitive competitors and ultimately leave resistant bacterial strains behind to reproduce as a consequence of the natural selection process. Antibiotics are being overused and overprescribed globally. Epidemiological research has demonstrated a direct relationship between antibiotic consumption and the emergence and dissemination of resistance in bacterial strains [12].

For the last two decades, the absence of novel antimicrobial drug discoveries has tipped the efficacy of currently available antimicrobial drugs. The infection prevention and control (IPC) and the stewardship program were previously used to control the AMR but have been proven impotent to limit the expense of resistant forms of GNB such as *Pseudomonas aeruginosa* and *Enterobacteriaceae*. In the south of Europe, many isolates of *P. aeruginosa* (GNB) have been identified as approximately 10-50% multidrug-resistant (MDR) [13]. Recently, the relative attractions of different IPC approaches for control of MDR bacteria recommended a combination strategy consisting of four components involving stewardship, source control, environmental cleaning, and standard care, which may demonstrate an efficient intervention [14]. However, these strategies have been involved in the prevention of the transfer of resistant isolates among humans but do not mark the horizontal gene transfer (HGT) between and within the species of microbes [15].

The current scenario concerning the control of AMR is unclear and cryptic. It is necessary to explore new classes of antimicrobial drugs with multiple mechanisms to create novel methods to overhaul the resistance and to turn up alternatives to antimicrobials that might help to abate AMR and ultimately noxious infections. The main kingpin of this article is to demonstrate the use of alternative therapeutics of antibiotics and cutting-edge methodologies to bridge the gap between early discovered drugs and clinical development, which might lead to diminished AMR. It is the need of the hour to probe new target approaches and synergistic policies at the national and international levels to surpass AMR.

## 2. Antimicrobial Discovery and Resistance

AMR is the supreme challenge in the treatment of infectious diseases, posing a dire public health risk on a global scale [16]. The pace of new antimicrobial discoveries is too murky that it was observed that even in 1987, no new classes of the drugs were given away [17]. The issue is made more complex by the collateral destruction from excessive use of antimicrobials when agricultural or clinical antibiotics are used, which produce selective pressure that causes AMR not only in the targeted pathogen but also in other microbes [18]. After the golden age of antimicrobial drug discoveries (1940 to 1960), there was an innovation gap from 1962 to 2000 during which no new antimicrobial class was presented [19]. Three logical reasons may clarify this gap: (i) research-associated troubles in obtaining fresh sources of broad-spectrum antibiotics against MDR pathogens that are nontoxic for humans, (ii) less appreciation of new antibiotics, and (iii) expensive processes or methods to develop the antibiotics [20].

Environmental and clinical AMR have been aggregating since the prevailing development of antimicrobials. Certainly, clinical resistance is recurrently sensed within years with the formation of novel antimicrobials [19, 21]. Archived soil survey via polymerase chain reaction has shown that the occurrence of environmental AMR genes has been on the upswing since 1940 [22]. Several isolates that are antimicrobial resistant, such as the deadly fluoroquinolone-resistant *P. aeruginosa* strains, MRSA, and vancomycin-resistant *enterococci* (VRE), have been gradually increasing since the 1980s [23].

## 3. Emergence and Spread of Antimicrobial Resistance

The main factors contributing to the incidence of AMR include microbial and human sources, excess clinical usage, public perception, agricultural and animal applications, commercial pressures, and vaccination reluctance [24]. Approximately, 1400 known human pathogenic species of bacteria, fungi, helminths, protozoa, and viruses have been reported on the earth [25]. The survival of this huge number of pathogens is due to their ability to adapt to environmental changes [26]. The collective adaptive response of microbes is the key to their prevalence. Their adaptive response includes a mutation in genetic material or the transfer of adaptive mobile genes between diverse bacterial populations [27, 28]. Under the persistent application of antibiotics, bacteria strive to evolve new strategies to confer resistance to antimicrobial drugs due to the increase in selective pressure, which ultimately contributes to the emergence of AMR. As far as the human population is proceeding to increase, the number of bacteria also increases similarly. Likewise, the urbanization of the human population provides an instant proliferation of infectious agents. Moreover, the ease of transport for humans also contributes to the transmission of infectious agents worldwide [29]. In this regard, a study instigated a novel iron-carbon microelectrolysis/electro-biocarrier-membrane bioreactor system for the removal of antibiotics from livestock water wastes [30].

The high efficacy of antimicrobial drugs to treat all disorders results in increased use of antibiotics, which ultimately builds up selective pressure on the bacteria. This selective pressure results in an increased AMR [31–34]. The inability to accurate diagnosis of infectious diseases by clinicians involves the inappropriate prescription of antibiotic drugs, but this can be controlled by advancing accurate diagnosis. In a Lebanese investigation, 52% of cases with inappropriate prescriptions and 63.7% with the wrong duration of time prescription from physicians were observed [35]. The serial application of drugs resulting from the patient's request and self-medication also exerts selective pressure on the microbes, which prompts AMR [36, 37]. The probability of the wrong implementation of antimicrobials is increasing over time leading to resistance development [38].

The use of antibiotics in agriculture to improve crop quality and yield is requisite to meet the increased demand for food. But, AMR genes also exist in the natural environment or agricultural lands, which may lead to the incidence of AMR as another contributing factor [32, 34]. The utilization of antibiotics in animals, especially in food-producing animals, is also a potent source of AMR spread. The resistant bacterial strains in food-producing animals are ultimately transferred to humans via direct or indirect content and consumption through sustainable agriculture-animal-human chains [39].

A range of different antimicrobials is used in society for cleanliness, ranging from floor cleaners to drops for the eyes and other objectives. This antimicrobial use replaces the other disinfection methods such as alcohol, autoclaving, and bleach, and this ultimately leads to the prevalence of AMR. Human sewage, poor sanitation, person-to-person contact, contaminated food, and water are also involved in the spread of resistant genes in the environment [40]. Today, a majority of people also supply a reservoir for pathogenic strains by decreasing the use of vaccines against diverse pathogens [41].

## 4. The Burden of Antimicrobial Resistance

AMR has become a global concern irrespective of the stage of income [7]. The mortal cost of AMR is very high and will progressively increase due to the lack of measures to harness the worldwide problem. It has been observed that patients with septic-resistant strains have a more hospitalization and mortality rate as compared to nonseptic patients. It was estimated that in the United State, two million people annually develop infections due to the evolution of new resistant microbes, consequently resulting in the deaths of more than 23,000 people. Similarly, in Europe, 25,000 deaths eventuate every year attributed to AMR. MRSA and resistant strains of *S. pneumoniae* are the principal contributors to mortality [42, 43]. In nondeveloping countries, the hazard of anti-microbial-resistant infections is even more stern. Recent reports suggest that resistance development against the treatment of TB, malaria, and retroviral diseases exhibits a massive negative impact on lower-income countries. The increasing frequency of antibiotic-resistant strains of TB is highly documented [39].

In 2013, 480,000 new cases were reported, of which most cases remained untreated [44]. Similarly, resistance to malarial treatment and antiretroviral therapy has been observed in numerous countries [45]. The economic cost related to the handling of these infections exceeds billions of dollars. Recently, O'Neill showed that globally 700,000 deaths were credited to AMR. However, O'Neill's research project counted the resistance rate in only three bacterial infections, MRSA, *E. coli*, and *Klebsiella pneumoniae* as well as the transmittable diseases, such as human immunodeficiency virus, TB, and malaria. If the present trajectory is not altered, it is expected that the worldwide number of deaths assigned to AMR would be 10 million, and economic burdens would be equal to the US $100 trillion per annum by 2050 [46].

## 5. Driving Forces in Generating Antimicrobial Resistance

The diffusion and development of antibiotic resistance are driven by three major forces, which are immune recognition and response, bacterial competition within communities, and exogenous antibiotic pressure [47]. Firstly, antibiotic resistance evolves in bacteria that may reside in the human body, as these bacteria want to procure them from the immune system of the host body [48]. Secondly, antibiotic resistance is driven by the intraspecies competition of the bacteria. For instance, the mutated strains of *S. aureus* (resistant) outcompete the wild strains through the release of Bsa (bacteriocin of *S. aureus*) and surfactant molecules. The selection of the resistant *S. aureus* strain along with the increased Bsa concentration thus causes more virulence [49]. Thirdly, the enhanced exogenous administration (pressure) of the antibiotics to a host stimulates the immediate development of antibiotic resistance genes (ARGs). ARGs are present in both environmental strains of bacteria as well as in the human body [50]. Mainly two distinct pathways, vertical evolution (gene mutations) and horizontal evolution (HGT between bacteria), are responsible for ARG acquisition; however, other factors, like the spontaneous resistance genes' presence in the environment, complicate furthermore this phenomenon [51, 52]. In a study, a diet containing the growth-enhancing antibiotic mixture was fed to pigs for around two weeks. When their microbiota was analyzed, they showed the enhanced expression of the ARGs, which also conferred resistance to drugs, even those that had not been administered previously [53]. This shows that ARGs are naturally present in the bacterial population, but their transmission is, however, stimulated by different driving forces. Further, antimicrobial resistance drivers are unhygienic drinking water, antimicrobial over and misuse, poor healthcare facilities and qualities, and unawareness about the medicines and vaccines [54–57]. All microbial species rapidly adopt the resistance mechanism and produce destructive effects. Hence, it can only explain the resistance development mechanisms in bacteria.

## 6. Mechanisms of Antibiotic Resistance

There are two types of antibiotic resistance present in bacteria. One is intrinsic in a class of bacteria comprising intrinsic determinants of resistance. The second one is an acquired resistance either by a mutation in genes or by gaining external genetic determinants of resistance from intrinsic-resistant organisms through HGT. In HGT, bacteria acquire foreign DNA/gene through three main mechanisms: (i) transformation which is the injection of naked DNA, (ii) transduction which is carried out with the help of phages, and (iii) conjugation in which transfer of genetic material occurs through mobile genetic elements that can be presumed to be transposons or plasmids [58]. Similarly, posttranslational modification has been turned up in the development of drug resistance [59, 60]. Another method involves the use of integrons, which are site-specific recombination systems having mobile genetic cassettes. Integrons offer a simple strategy to incorporate resistant genes into the chromosome of bacteria, and they also provide an option for the transfer of machinery that will be used in the expression of these genes [61].

**Figure 1:**
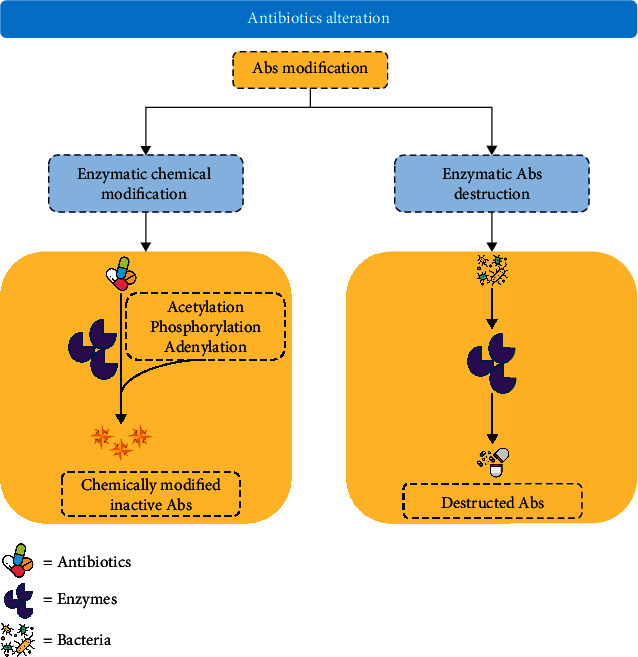
Schematic diagram showing the enzymatic alteration of antibiotics producing multidrug resistance (Abs = antibiotics).

**Figure 2:**
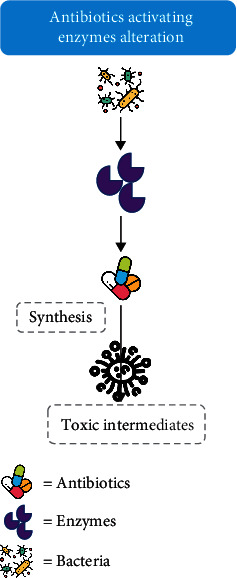
Schematic diagram showing the alteration of antibiotics activating enzymes that produce toxic intermediates that contribute to multidrug resistance.

**Figure 3:**
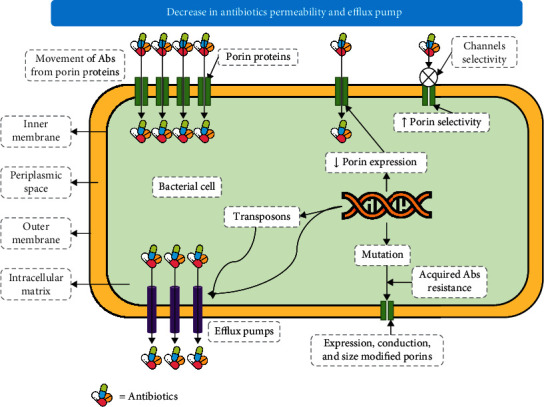
Schematic diagram showing a decrease in antibiotic permeability and efflux pumping of antibiotics (Abs = antibiotics).

**Figure 4:**
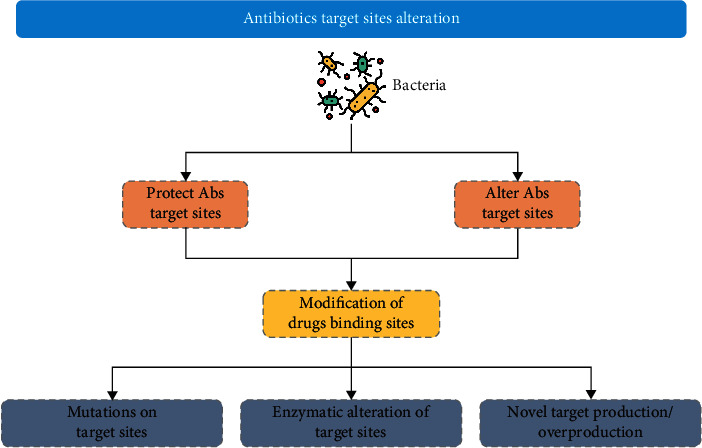
Schematic diagram showing alteration of antibiotic target sites in multidrug resistance (Abs =antibiotics).

In microorganisms, resistance is achieved through multiple pathways or mechanisms based on biochemical routes; thus, it can be classified into alterations of the antibiotic molecules (Figure 1) [62, 63], alteration of antibiotic activating enzymes (Figure 2) [64], decrease in membrane permeability and efflux pump activity expression (Figure 3) [11, 65–68], and alteration in antibiotic active sites (Figure 4) [69–74].

## 7. Cutting Edge Methodologies to Manipulate Antimicrobial Resistance

**Figure 5:**
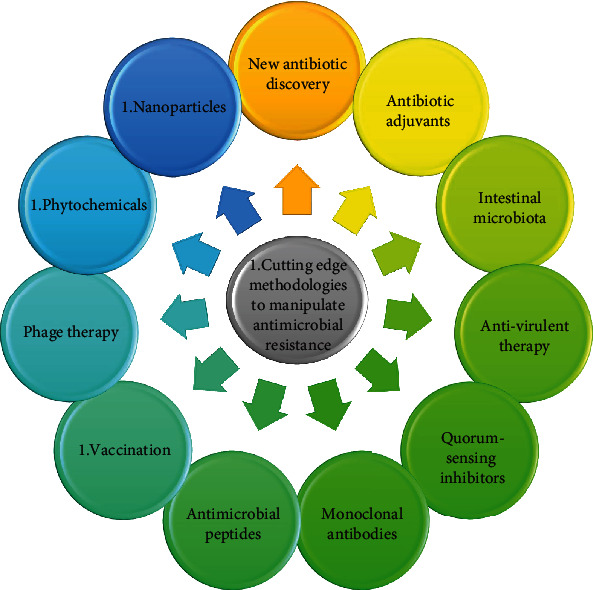
Showcasing different cutting-edge methodologies that manipulate antimicrobial resistance.

To cope with AMR, currently, several approaches and/or strategies are on their way (Figure 5). Several so-called promising alternatives have been studied for decades without any practical impact. Among these, the most promising ones have been reviewed as follows:

### 7.1. New Antibiotic Discovery

**Figure 6:**
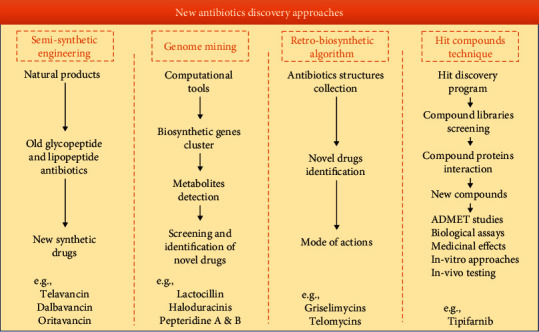
Schematic diagram showing the approaches for new drug discovery against multidrug resistance.

The major obstruction with the new antibiotic discovery includes a prolonged process of drug production and cost expensiveness. The present situation reflects that a new compound that was discovered in the laboratory (having promising activity) would take approximately 15 years for selection and usage as a therapeutic agent [75]. Therefore, researchers strive to modify or rediscover the old drugs instead of discovering new drugs or antibiotics [76].  Figure 6 demonstrates the approaches for new drug discovery against multidrug resistance.

#### 7.1.1. Semisynthetic Engineering

A semisynthetic engineering approach is used to produce new drugs with improved pharmacokinetics (PK) and broad-spectrum activity [77]. In this approach, old glycopeptide and lipopeptide antibiotics (derived from the natural product) are altered using various enzymatic and chemical methods to manufacture new semisynthetic drugs [78]. Utilizing a semisynthetic approach, three vancomycin derivatives such as telavancin, dalbavancin, and oritavancin have been produced [79]. Moreover, the easy modification of vancomycin C-terminus has led to the production of various novel semisynthetic drugs [80]. Other antibiotics that have been successfully synthesized or modified include azithromycin and clarithromycin (erythromycin derivative), minocycline, doxycycline (tetracycline derivative), rifampicin, and tigecycline (rifamycin derivative) [78].

#### 7.1.2. Genome Mining Technique

A genome mining technique has been used for novel antibiotic discovery. This biosynthetic gene cluster (BGC) guides the isolation of drugs. Computational tool kits like anti-SMASH and PRIS are mostly used for the identification of BGC [81]. Extensive studies have reported a list of strategies for the discovery of novel drugs with genome mining. The strategies comprise ribosome engineering, constitutive promoters' insertion mediated by CRISPR-Cas9, culture condition optimization, genetic manipulation of transcriptional regulators, heterologous expression, epigenetic control perturbation, and small molecule elicitor use [82–85]. Lactocillin (made up of ribosomally encoded and posttranslationally modified peptides: RiPPs) specific for the treatment of Gram-positive bacterial infections was manufactured by using the genome mining technique. The BGC for this antibiotic complex was identified in *Lactobacillus gasseri*. Halophile is another example of the RiPP antibiotic complex; its BGC was isolated from Gram-positive bacteria [86]. Taromycin A (similar to daptomycin structure) is a nonribosomal halogenated lipopeptide antibiotic that was also generated by the genome mining of *Actinomycetes*. This antibiotic was isolated by the introduction of a biosynthetic pathway clone into a heterologous host [87]. Park et al. reported that *cis*-amide acyl-side chains functionalized new pteridine metabolites, named piperidine A and B, by the genome mining of *Photorhabdus luminescens* (nematodes associated with gammaproteobacterium) [88]. Genome mining identified UCS1025A pyrrolizidinone and terpenylated diketopiperazines in *Myceliophthora thermophila* fungus and *Streptomyces youssoufiensis* OUC6819 [89, 90]. A recent approach named high-throughput elicitor screening (HiTES) introduces reporter genes into the BGC for the rapid reading of gene expression and screens the libraries of small molecules to identify candidate elicitors. This approach revealed a novel lanthipeptide metabolite in *Saccharopolyspora cebuensis* [91], 2 novel metabolites in *Streptomyces hiroshimensis* [92], and 14 novel metabolites in *Streptomyces albus* J1074 [93]. Studies have reported growth inhibitory activities of novel discovered metabolites against GNB [91, 92].

#### 7.1.3. Retro-biosynthetic Algorithm and Hit Compound Technique

Recently, a retro-biosynthetic algorithm has been defined. It is applied to a large collection of antibiotic structures for the identification of a novel drug following a new mode of action [94]. For example, using this method, griselimycins and telomycins are synthesized that insert their activity by interacting with DNA clamp proteins like DnaN and membrane cardiolipin phospholipids, respectively [95]. Similar to these techniques, other computational strategies have focused particularly on identifying novel antimicrobial compounds, for instance, the hit compound technique. In this technique, hit discovery programs identify a set of defined chemical structures (hit compounds) in the compound libraries that produce activities against microbial targets. Although the cytotoxicity and selectivity of the hit compounds are required to be investigated, in this regard, ADMET studies, medicinal effects, and biological assays confirm the hit-to-lead optimization and guide the structural improvements of the hits against pathogens. Sequentially, hits are then tested in whole-cell, isolated, or exposed in vitro approaches and later in infectious animals to evaluate their chemical identity, integrity, and pharmacodynamic and pharmacokinetic properties. [96]. The latest study applied this approach to identifying antiviral drugs, which could be applied to other microbes as well. Around potential repurposed 15 drugs' interactions with the main protease and 23 drugs' interactions with RNA-dependent RNA polymerase were probed. Among them, tipifarnib, emodin, and omipalisib showed activities in Calu-3 human lung cells [97].

A community for open antimicrobial drug discovery (CO-ADD) is an open-access facility that screens compounds submitted by any chemist for their antimicrobial efficacy. CO-ADD is a community-based approach that uses and discovers new antibiotics [98]. This approach has identified various active compounds that are hoped to be used as antibiotics in the coming future to decrease the burden of AMR [95]. Additionally, two organizations such as CARB-X and IMIENABLE have been made, which provide specialized expertise and funding to accelerate the process of solving the superbug crisis of AMR [75]. However, there are only a few drugs against the GNB, which shows that it is quite difficult to treat a wide range of all bacterial species. Therefore, there is a need for hours to invent more effective formulas at a broad-spectrum level. Nevertheless, it is questionable how long all these newly discovered antibiotics would stand against the resistance mechanism. Researchers are trying to find alternative ways to combat AMR. Currently, numerous promising approaches have been presented and are on way to fight against AMR through novel discoveries.

### 7.2. Antibiotic Adjuvants for the Inhibition of Resistance

Along with the attempt to discover new antibiotics, it is also vital to preserve our existing drugs [99]. However, a strategy that can be used for the preservation of the existing drugs has introduced the use of antibiotic adjuvants. These adjuvants are used not only to block resistance but also to improve the efficacy of existing drugs [100, 101]. Antibiotic adjuvants are mostly used in combination therapy [102, 103]. Adjuvant therapeutics showed response by (i) modulating active transport, (ii) increasing drug absorption, (iii) modulating drug transformation to the intestine or liver, (iv) enhancing immune activity, and (v) decreasing the rate of elimination [104]. Antibiotic adjuvants are generally classified into two categories: class I adjuvants and class II adjuvants. Class I adjuvants are further divided into two classes: class I-A adjuvants and class I-B adjuvants. Their different mechanisms of action are mentioned in Table 1.

The best-known antibiotic combinations are aminoglycoside and penicillin to treat enterococcal infections. This combination therapy works far better because synergistic interactions are accessed, and the drug efficacy seems more in this case rather than in a single drug. As a result, bacteria are killed at a faster rate, and hence, resistance is also blocked [105]. Antibiotic adjuvants do not show any effect when utilized alone; rather, these enhance the antibacterial activity of the drugs when used in combinations [106]. In this new era of antibiotic resistance, antibiotic adjuvants offer a promising approach to overcoming antibiotic resistance, either by direct blockage of resistance or by enhancing the effect of other antibiotics. Therefore, it is the need of the hour to pay attention to exploring new adjuvant antibiotics to address the emergence of resistance [105, 107]. Borselli et al. reported the potential activity of polyamino-isoprenyl derivatives with florfenicol. The molecules inhibit efflux pumps by collapsing the proton-motive force (PMF) caused by the induction of inner membrane depolarization [108]. Another study investigated the combined effect of farnesyl spermine compound 3 with minocycline and doxycycline and resulted in a substantial decrease in antibiotic resistance in *P. aeruginosa* [109]. Sometimes, the coating of compounds can also enhance the antimicrobial activity of drugs. For instance, Wang et al. pegylated the azelaic acid that enhances its antimicrobial activity [110]. Recently, a study developed computer-guided antimicrobial foldamers. They coadministered the foldamers with antimicrobial peptide PGLa, which substantially reduced the MDR in *K. pneumoniae*, *Shigella flexneri*, and *E. coli* [111].

### 7.3. Intestinal Microbiota: A Battlefield for Combating Multidrug-Resistant

**Figure 7:**
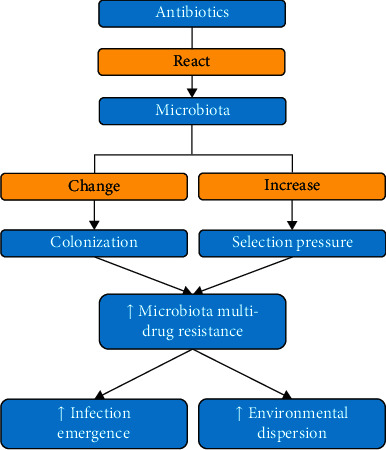
Schematic diagram showing the development of multi-drug-resistant microbiota due to antibiotics.

The use of intestinal microbiota is a better choice in an attempt to decrease MDR. It is noted that the intestinal microbiota of animals remains resistant to the constant occupation of exogenous bacteria. It is known as colonization resistance (CR) [119]. The introduction of antibiotics produces a conflicting reaction in the microbiota. It causes a change in CR and an increase in the selection pressure. Both of these events increase the development of MDR bacteria in the gastrointestinal tract (GIT) [120]. It is noted that an abundance of intestinal MDR bacteria would result in an increased emergence of infections and dispersion in the environment [121].  Figure 7 explicates the effect of antibiotics on the development of MDR in microbiota. Accordingly, conserving the microbiota in contradiction to antibiotics may favor combating the MDR bacteria. The influence of antibiotic drugs on microbiota for the acquisition and development of MDR species continues to be reviewed in a call to assist clinicians to select the more approved type of drugs for treatment [122].

**Figure 8:**
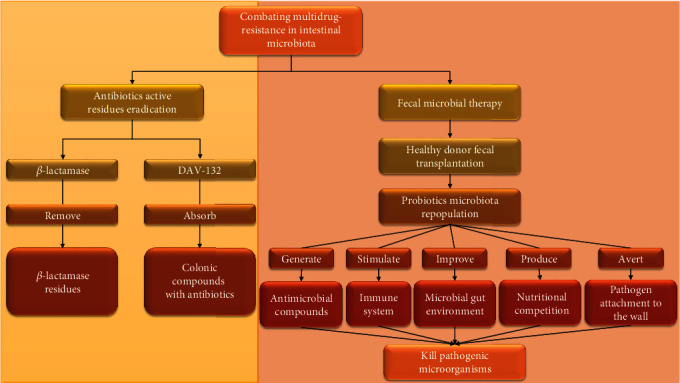
Schematic diagram showing the eradication of multidrug resistance in intestinal microbiota.

One method for protecting the microbiota from antibiotic drugs is to eradicate the active residues of antibiotics in the colonic place, where intestinal bacteria are present in high amounts. Two possibilities are currently being studied: (i) the use of beta-lactamase for decomposing the *β*-lactam residues present in the gut. Now utilization of phase II SYN-004 testing (in which orally supplied *β*-lactamase is produced by Rockville) has been revealed to eradicate the ceftriaxone drug that remains present in dog and pig GIT [123]. On the other hand, (ii) utilization of DAV-132 (recognized by Da Volterra Co., Paris, France) in phase II is used for adsorbing freely moving colonic compounds along with antibiotics [124] for future protection and development of microbiota (Figure 8). The second method is fecal microbial therapy (FMT). It has been working for decades to treat various intestinal infections. Although it showed varied results [125], FMT therapy is used, especially in China, for the treatment of *Clostridium difficile* infections (CDI) and other enteric diseases. Recently, a paper has renewed clinical attention to FMT by showing its forceful activity against persistent CDI. FMT involves the utilization of fecal material from the healthy donor (pathogen-free) to a recipient for the repopulation of microbiota probiotics present in the gut. The probiotics are capable to kill pathogenic microorganisms by (i) generating antimicrobial compounds, e.g., organic acids and bacteriocins; (ii) stimulating the immune system of the intestine; (iii) improving the gut microbial environment; (iv) averting the attachment of pathogens to the wall; and (v) producing competition with them for nutrients with an increased rate of digestion and nutrition absorption from the intestine (Figure 8). FMT may also increase the risk of pathogen transmission. So, proper screening and donor selection are prerequisite with intensive care. This transmission could also be minimized by autologous FMT, which involves the usage of patient fecal material that has been well reserved previously (before the administration of antibiotics and utilization of semisynthetic or synthetic microbiota) [125, 126]. The frequently used probiotics are *Lactobacillus*, *Bacillus*, *Enterococcus*, *Lactococcus*, *Bacteroides*, *yeast*, *Bifidobacterium*, *Pseudomonas*, *Trichoderma*, *Pediococcus*, and *Aspergillus*. These have been reported to be used effectively to kill intestinal pathogens [127]. However, advanced studies are focusing on discovering the specific bacterial species and precise chores for the release of MDR bacteria from the intestine.

## 8. Prioritized Other Modern and Novel Alternative Therapies

### 8.1. Antivirulent Therapy/Quorum-Sensing Inhibitors

**Figure 9:**
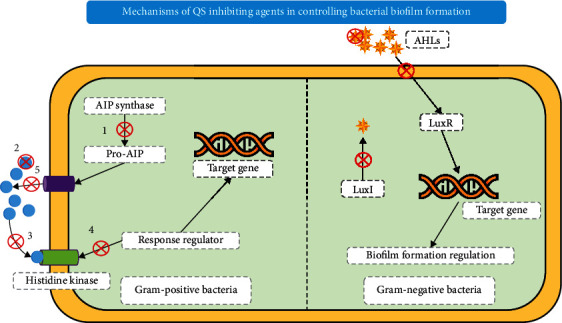
Schematic diagram showing inhibition mechanism of quorum sensing to control the formation of bacterial biofilm. The diagram shows the mechanisms by which QS inhibiting agents control bacterial biofilm formation marked with numbers: (1) inhibit the synthesis of AIs; (2) reduce or inactivate the effect of AI by oxidoreductases, AHL-lactonases, antibodies, etc.; (3) using AI antagonists that interfere with signal receptors; (4) disrupt the signaling cascade by interfering with the response regulators; and (5) by blocking AI efflux, inhibition of cell-to-cell signaling, and reduction of the accumulation of extracellular AIs.

Antivirulent therapy purposes a depletion of bacterial toxicities without declining the development of pathogens by using quorum-sensing (QS) inhibitors [128]. Prokaryotic organisms use the QS for cell-to-cell communication when they are present in high concentrations, resulting in triggering prokaryotic adaptive immunity. So, by using QS inhibitors, adaptive immunity could be suppressed, which results in nonpathogenicity [129, 130]. The automatic mechanisms through which QS produces an operative response in Gram-positive bacteria and GNB are different from each other, i.e., Gram-positive bacteria exploit oligopeptides, while GNB does N-acyl-L-homoserine lactones. Quorum quenching is carried out in three ways, including sequestration, competition, and destruction of signal.  Figure 9 demonstrates the inhibition mechanism of QS inhibitors to control the formation of bacterial biofilm.

The QS inhibition system is considered more advantageous over conventional antibiotics because it involves disruption of communication mechanisms without damaging the individual cells. Hence, this approach produces less selective pressure and decreases the degree to which AMR is produced during treatment. Using antivirulent therapy, a large number of investigations have been performed on diverse lethal pathogens such as *S. aureus*, *P. aeruginosa*, *Vibrio*, *Cholera*, *E. coli*, and *Vibrio fischeri* [39]. First-time QS inhibitors were collected from Delisea pulchra (red marine algae) and Ascophyllum *nodosum* (brown algae) [131]. Later on, extracts of various plants, for instance, Dalbergia *trichocarpa*, *Syzygium aromaticum*, *Terminalia chebula*, *Moringa oleifera*, and Conocarpus were used against *Chromobacterium violaceum*, *Chromobacterium violaceum*, *P. aeruginosa*, and *E. coli* strains as QS inhibitors [132]. Currently, furanone, cis-2-dodecenoic acid, lyngbyoic acid, meta-bromo-thiolactone, eugenol, iberin, PD12, 6-gingerol, ajoene, and azithromycin (AZM) antibiotics are used mostly for QS purposes [27, 100, 133]. Zhong and He in their minireview found two approaches, quorum quenching (QQ) enzymes and quorum sensing inhibitors (QSIs), that reduce the bacteria's virulence and drug resistance pressure by inhibiting the QS and biofilm formation in bacteria [134].

### 8.2. Passive Immunization (Monoclonal Antibodies)

**Figure 10:**
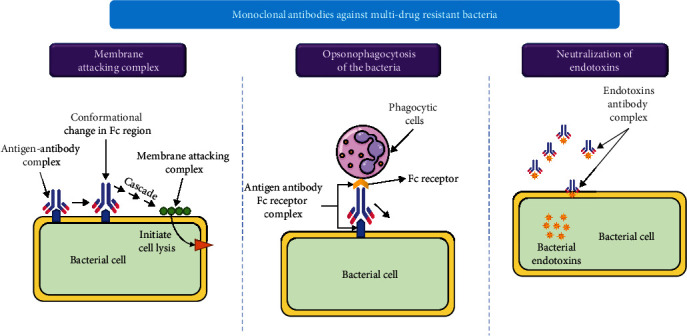
Molecular mechanism of monoclonal antibodies against multiresistant bacteria.

Monoclonal antibodies (mAb) are contemplated as potential agents to kill the bacteria since the first treatment using sera. mAbs have been produced from variable sources like mice, rabbits, and chimpanzees They were first proved unsuccessful due to the formation of autoantibodies and short half-life or life span. Now, humanized mAbs have been synthesized which stop, or at least decline, the development of immunogenicity [20]. The mechanisms through which mAbs play a role in infectious diseases against pathogens are the following: neutralization by the complement-mediated response, removal of bacterial exotoxins, and induction of antivirulent antibodies or direct bactericidal production for killing the bacteria [135].  Figure 10 shows the mAb actions against MDR bacteria [136]. Similar to phage therapy, the major gain of using passive immunization over antibiotics is its specific targeting, so mAb is designed against their target pathogen. mAbs have been used in clinical trials in combination with different antibiotics. In conjugated form with antibiotics, they eliminate intracellular pathogens or sometimes prove to be enough alone to treat different diseases, which may include pneumonia, sepsis, diarrhea associated with *C. difficile*, cystic fibrosis, and burns [137, 138].

The bezlotoxumab was the 1st antibacterial mAb that accessed the market and has been appearing to be effective to combat the reappearance of CDI (when associated with placebo) [139]. Recently, Skurnik et al. observed that Alopex (F598) targeting PNAG was effective to defend the mice against MDR *P. aeruginosa* and *Enterobacteriaceae* [140]. In the last ten years, collaborative work was done to treat infections caused by *S. aureus* (like pneumonia) via clinical trial phase I-III with mAbs. These were used against the clumping factor A, GrfA, alpha toxin, leukocidins, and poly-b-1,6-N-acetylglucosamine. However, the majority of the mAbs failed to show efficacy, and only a few such as Salvecin, MED14893, and ASN100 have been proven successful in clinical development [141]. Likewise, for the treatment of *P. aeruginosa* infections, antibodies like panobacumab, Aerucin, and MEDI3902 are being utilized against the O-antigen carbohydrate and type III secretion system to treat nosocomial pneumonia [135]. Zurawski and McLendon described several studies on the use of mAb against resistant bacteria. For instance, the antibiotic conjugate (TAC) RG7861 (anti-*S. aureus* TAC, DSTA4637S) was found in the conjugated form of mAb with antibiotics and was used against *S. aureus* [142]. Certainly, omics technologies have also been used for the development of mAb against resistant microbes employing the methods of epitope identification with genomics, transcriptomics, proteomics, and metabolomics databases. The epitope selection includes major circulating isolates, conversed levels of amino acids, exposed surfaces, virulent antigens, and expression at the infection level. Such types of approaches can assign to high-value epitope identification and effective mAb development in bacterial cloning [143].

### 8.3. Antimicrobial Peptides

**Figure 11:**
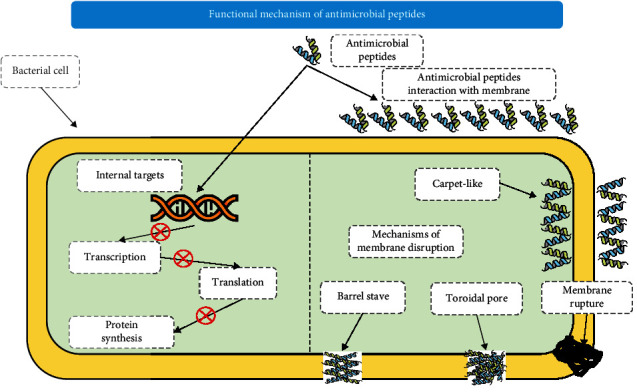
Functional and molecular mechanism of antimicrobial peptides against multi-drug-resistant bacteria.

Antimicrobial peptides (AMPs) are typically small, amphiphilic, cationic molecules. These are produced from fungi, bacteria, plants, and all vertebrates including humans [144]. They show anonymously broad-spectrum activities against GNB and Gram-positive bacteria, fungi, yeast, protozoa, parasites, and viruses. They are used as potential and nifty therapeutic agents [145]. These peptides are structurally different from each other and composed of ribosomal proteins (usually >100 amino acids) or some nonribosomal compounds (enzymes) [146]. The mechanism of action through which AMPs affect microbes involves contact with the surface membrane to produce a hole in the membrane. It moves across it to disturb different cytoplasmic processes, including inhibition of some enzymes, cell division, macromolecular synthesis, or stimulation of autolysis.  Figure 11 depicts the functional action of AMPs combating MDR.

However, if we talk about AMR development, then peptides show slower and or less resistance produced as compared to antimicrobial drugs. It is because of three basic reasons: (i) structural diversity of peptides with the surface membrane, (ii) obligatory interaction of AMPs, and (iii) potential of AMPs having multiple targets; thus, making eradication of any of the targets does not produce resistance [147]. Moreover, it is observed that like antibiotics, AMPs do not involve the production of mutagenesis [148]. But the negative point is that AMPs are expensive to produce and have a short half-life. New technologies for AMP formulation, modification, and distribution have been studied to overwhelm the inadequacies of bioavailability, PK, cost-effectiveness, and toxicity [149]. For increasing bioavailability, computer-assisted AMPs are recently being proposed [150]. Moreover, alteration of peptide structure in peptidomimetics structure is proteolytic resistant; thus, it is a talented approach to revamp the pharmacokinetic properties of peptides [100, 151, 152].

The energetic strategy to enhance the AMP's activity is the encapsulation process in which liposomes, hydrogels, polymeric structures, carbon nanotubes, nanocapsules, and DNA cages are used to decrease collateral damage and metabolic degradation [153]. Moreover, a selective target, a wide spectrum, lower toxicity, and a different mechanism of action make peptides a superlative option for future medicine [144]. Recently, antibiotics are being used in combinations with AMPs to boost their efficacy against microbes and result in less development of AMR. PR-39 and lactoferricin B, Bac7 and lactoferricin B, ampicillin, streptomycin, and Nisin Z are examples of synthetic combinations of antibiotics and AMPs. They have been proven to be more effective against MRD bacteria [154, 155].

One considerable advantage of AMPs is having the ability to neutralize endotoxemia or sepsis. That is a common and unsafe complication of systemic antibiotic therapy. In addition, certain peptides play an effective role in mammalian innate immunity [147]. One of the supreme roles is their potency to stimulate the response of the innate immune, while simultaneously reducing the possible harmful inflammatory response [156]. For example, a synthetic peptide IMX00C1 has been exposed to be defensive in bacterial infectious diseases in animals [147]. Mwangi et al. listed several AMPs that have substantial effects on MDR [157]. It has been reported that multiple AMPs such as teixobactin, pyrrochorrycins, apeadicins, oncocins, omadacycline, and aminomethylcycline have MDR activities against Gram-positive bacteria and GNB [158]. Around fifteen studies have reported promising results of AMPs against MDR *H. pylori* [159].

### 8.4. Vaccination

**Figure 12:**
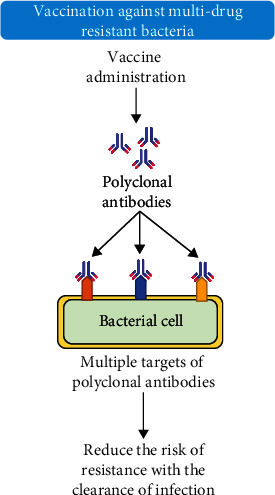
Schematic diagram showing the action of vaccines combating multi-drug-resistant bacteria.

Present vaccines support reducing the problem of AMR. Resistance is not a clinical obstacle for any of the infectious bacterial diseases for which we systematically vaccinated for decades like pertussis, and diphtheria (because they are hardly seen and infrequently treated). The most noticeable potential benefit of vaccinations is the possibility of decreasing infection rates caused by bacteria that are difficult to treat and have a high mortality rate. Vaccine-based strategies reduce the need for prescribing antibiotics. It results in less or no selective pressure, a basic cause of the emergence and transmission of the resistant gene. This idea is reinforced through investigations that a decrease in the medical use of antibiotics is accompanied by fewer resistance rates. On the other hand, all current drugs have no such potential as vaccines. Moreover, the immune reaction is raised by vaccination, so specifically targeted vaccines are being utilized against particular microorganisms. These may have little effect on humoral bacteria of the body [160]. The introduction of the Hib conjugate vaccine proved to be effective to eliminate bacteremia, pneumonia, and Hib meningitis. It showed not only a reduction in the usage of antibiotics but also prevents the evolution pattern of resistance development [161].  Figure 12 denotes the action of vaccine and polyclonal antibody production in combating multi-drug-resistant bacteria [29].

In the United States, a seven-valent pneumococcal conjugate vaccine (PCV7) is used for the treatment of invasive pneumococcal disease (IPD), resulting in an 84% reduction in the rate of MDR-IPD [162]. It has been estimated that PCV and Hib conjugate vaccines could reduce antibiotic use for these certain diseases by 47% [163]. Similarly, respiratory virus vaccines are produced to combat the resistant influenza virus. These communicate diseases to a nonsignificant proportion of people each year [164]. These vaccines prompt a reduction in the influenza virus and could lead to a curtailment in selection pressure caused by antibiotic treatment of influenza [165].

In certain cases, vaccination causes the elimination of a particular pathogen and decreases the usage of a broad-spectrum antibiotic (empirical treatment) against clinical conditions, i.e., pneumonia. In this way, through immune-based strategies, the number of resistant infections can be decreased [166]. Different types of vaccines such as formalin-inactivated vaccines, bacterial ghost vaccines, cell component vaccines, bacterial polysaccharide vaccines, subunit vaccines, recombinant vaccines, and DNA/RNA vaccines have been developed [167, 168]. Many studies have reported the use of vaccines against MDR microbes, for instance, typhoidal *Salmonella* [169, 170], *Acinetobacter baumannii* [167], tuberculosis [171], *E. coli* and *Salmonella* (Sabry Abd Elraheam [172]), cholera and traveler's diarrhea, *Enterobacter* spp., *P. aeruginosa*, *S. aureus*, *A. baumannii*, *Campylobacter* spp., *H. influenzae*, and *S. pneumoniae* [173].

### 8.5. Phage Therapy

Bacteriophages act as biocontrol to combat AMR. During the golden age of antibiotics, phage therapy was overlooked to control bacteria. However, the development of AMR has renewed the interest in phage therapy [174]. In phage therapy, specific bacteriophages are used as a potential alternative to antibiotic drugs [175]. Phages that carry only lytic cycles are used as biocontrol. This is because they cause pathogen lysis as compared to lysogenic phages, which may themselves be involved in anti-microbial-resistant genes spread instead of their elimination [176]. In lytic phage, cell lysis turns up by blocking the peptidoglycan synthesis or its cleavage via the lysine system [177]. Bacteriophages actively identify specific pathogen receptors, inject their DNA, multiply, and discharge their progeny via host cell lysis [178]. Phages specific to polysaccharide targets are used to eliminate the encapsulated bacteria present in the biofilm. Live phages may also produce an effective immune response in this case. Live phages are usually practiced against infectious strains like *Enterobacter*, *Shigella*, *E. coli*, and *K. pneumoniae* [179]. In dairy products, live phages are used to eliminate Lactobacillus. Several scientific reports have been reported on the treatment of infectious respiratory and systemic diseases, where the effectiveness of phage therapy has been observed [180].

**Figure 13:**
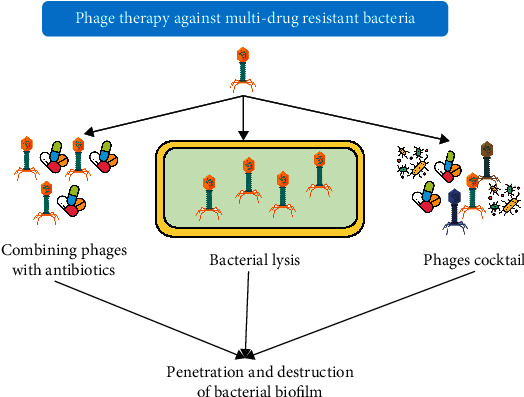
Schematic diagram showing the molecular action of phage therapy against multi-drug-resistant bacteria.

Bacteriophages such as JG024, MPK1, PAK-P1, M4, E79, PaP1, MPK6, PIK, and LUZ7 have been isolated against *P. aeruginosa* to treat urinary tract infections [181]. In Eastern Europe, bacteriophage therapy has been broadly used for therapeutic purposes with a positive history. However, this therapy has not been characterized in terms of efficacy, immunization, safety, selection of resistance, PK, and tolerance, perhaps due to fewer publications transferred from the East to the West [182]. The advantages of phage therapy over antibiotics have been evinced from several studies. For instance, phages effectively kill GNB and Gram-positive bacteria including (i) antibiotic-resistant strains and MDR organisms; (ii) there were no serious side effects because of being targeted for particular bacteria. This shows that phages multiply rapidly at the location of infection and have no side effects on the normal flora; (iii) the rate of resistance development is lesser as compared to antibiotics [183].  Figure 13 depicts the action of phage therapy against multi-drug-resistant bacteria [184]. Phage preparation is cost-effective, easy, and fast [185]. However, the effort is requisite for bacteriophage isolation and genetic modifications [178, 186].

Genetically modified phages were produced against *E. coli* that multiplied and killed the bacteria without cell lysis. In this way, inflammatory effects are minimized via less release of endotoxin as compared to the antibiotics and live lytic phages [187]. Still, phage therapy will not completely replace antibiotics, because it has some intrinsic disadvantages, such as bacteriophages cannot be taken intravenously due to the active immune response of the host cell showing no effectiveness against deeply present intracellular pathogens. Therefore, phage therapy can be directed to such infectious diseases where bacteria accessibility is easy, such as pneumonia and wounds. Moreover, phage therapy has some controlling and storage problems that need to be resolved, as there is no proper framework for its regulation [188]. In vitro conducting tests have revealed that controlled bacteriophages could kill MRSA selectively as compared to methicillin-susceptible counterparts [189]. Hagens et al. and Torres-Barceló et al. have proven that combined therapy using antibiotics and bacteriophages shows a more promising response to kill the pathogens than using an antibiotic and phage alone. This type of treatment is used to enhance the efficacy of an antibiotic drug against *P. aeruginosa*, *E. coli*, and MRSA [181, 190].

Major obstacles with the use of phages include biocontrol specificity for target bacteria and the existence of not any broad-spectrum phages. Hence, it prevents the use of a single individual bacteriophage against multiple strains involved in a single infection [191]. However, in neoteric efforts, phage cocktails are being designed to eliminate a high number of bacteria present in a system. In cocktail phage, if one phage is inhibited, then the second phage plays role in targeting the same pathogen more actively. The perfect preparation of the cocktail is obligatory because each phage exhibits a specific recognition pattern for cell wall receptors. For reducing the Pseudomonas load, a cocktail having six phages was used as a biocontrol [182]. Similarly, phage cocktails Listex (MICREOS) and List Shield (Intralytix, Inc.) have been constructed to inhibit the Listeria present in food [192]. Now, it has been preferred to use phage endolysin in place of living phage to target the bacteria. Hence, a recombinant preparation such as N-RephasinSAL200 (established by iNtRON Biotechnology, Seoul, South Korea) is being used to target staphylococcal infections [193]. Phage therapy has recently been reported against MDR urinary tract infections and found that phage therapy not only does bacterial lysis but also inhibits biofilm formation. Phages can easily penetrate the biofilm by producing or inducing the polysaccharide depolymerase and degrading the biofilm [184]. A study highlighted the risk of the development of resistance in a single phage and suggested the use of multiple phages (phages cocktails) [194].

### 8.6. Algae-Mediated Treatment

Domestic and agricultural wastewaters have substantially contributed to the generation of antibiotic-resistant bacteria and genes. This unfortunate condition results from the widespread and haphazard use of antibiotics for the sake of health applications in humans and animals [195]. Many antibiotic-resistant bacteria and antibiotic-resistant genes are found in most wastewater treatment plants (WWTPs) since all domestic wastewater eventually goes there. To remove traditional pollutants (e.g., dissolved organics, suspended solids, and nutrients) from WWTPs in the US and worldwide, conventional treatment processes are used, namely, preliminary, primary, and secondary treatments. Activated sludge (AS) is the most common secondary treatment process, whereby bacteria biodegrade dissolved organic carbon in wastewater [196]. Several methods, such as UV254, ozonation, and chlorination, have been reported to degrade or remove antibiotic-resistant genes [197–200]. Notably, microalgae are found abundantly in the aquatic environment and have a higher tolerance and removal ability to contaminants than bacteria. Because of their excellent capability to remove nutrients, heavy metals, and pathogens, microalgae are of significant importance to maturation ponds, as well as domestic or facultative ponds that treat small- to medium-scale municipal wastewater [201]. Compared to other algal species, green algae are more susceptible to antibiotics than nontarget organisms [202].

Even though there were many practical applications of green algae for antibiotic treatment, previous studies mainly concentrated on the removal efficiency of the target compounds. In addition, algae characteristics during treatment are poorly characterized. Second, an excellent removal capacity is not worthwhile [202, 203]. Ecofriendly biotechnology should be efficient at removing waste and have a low environmental impact overall. It must examine whether green algae exerted similar selective pressure on antibiotic-resistant bacteria. Typical biological processes are thought to be contributing to the selection of antibiotic-resistant bacteria and resistance transfer among bacteria. The target antibiotic was bacteriostatically tested before and after the algal treatment. Meanwhile, previous studies have shown that antibiotics can negatively impact nontarget organisms. The aquatic impact of the target antibiotics and corresponding effluent after the algal treatment was also assessed using rotifers to exclude the possibility that the toxicity of the target antibiotics increased after the algal treatment (as a result of UV irradiation or chemical degradation) [202, 204].

Numerous studies have reported algae-mediated treatments for the removal of antibiotic-resistant genes. Grimes et al. concluded efficient reduction in the pEX18Tc plasmid transformation, complete reduction of plasmid transformation, and 67% reduction in the transformation of plasmid in autoclaved algae [205]. In this context, a study confirmed the removal of induced ciprofloxacin resistance with freshwater algae treatment [206]. Another study compared the conventional wastewater treatment system with the algal-based system to remove antibiotic-resistant genes and bacteria. They found a substantial reduction in the sulfamethoxazole- and erythromycin-resistant bacteria in the algal-treated system than in conventional systems [196].

### 8.7. Phytochemicals

**Figure 14:**
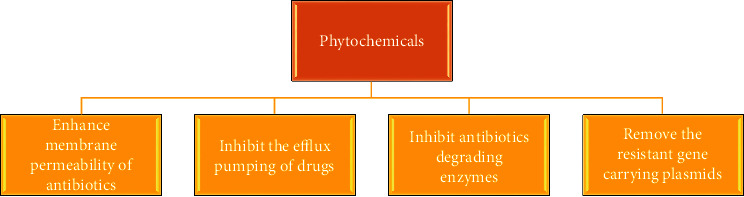
Actions of phytochemicals against multi-drug-resistant bacteria.

Combination therapy consisting of botanical and nutritional approaches can be effectively used for combating many infections, for instance, phytochemicals, flavonoids, isoflavonoids, and many other phenolic compounds [207]. The plant extract can also be used as an influential tool to defeat the resistance development mechanism in microbes [208]. The pathogens are not capable of easily training the resistance against phytochemical complexes obtained from different plant extracts; therefore, these can be used as an alternative to antibiotics [209]. The mechanism through which plant secondary metabolites such as alkaloids, flavonoids, quinones, terpenes, coumarins-lectins, and polypeptide tannins kill the microbes involves several strategies. It may include the disruption of the cell membrane or through decreasing the permeability of the membrane and enhancement of the influx system, blockage of genome synthesis, alternation in the structure of adhesion proteins and membrane-bound enzymes, and interference with cellular processes like the coagulation of cytoplasm and inhibition of QS (Figure 14) [210–212]. Additionally, these natural products have been involved in modifying protein-protein interactions; therefore, they can be used as effective modulators of mitosis, signal transduction, and immune response [213]. Hence, in a world of rising AMR where the selection of antibiotics shows no response, phytochemicals can be used to enhance the immune response to kill the bacteria [214]. The extract of babassu mesocarp, Aloe vera, and *Chenopodium ambrosioides* testified to increase phagocytic activity of macrophages. Similarly, Panax ginseng saponins and *Emblica officinalis* have been proven to enrich T and B cells to enhance innate and adaptive immunity, respectively [67, 215].

The crude extract consists of multiple components that produce a more profound and effective response against pathogens because they act not only to kill the microbes but also to produce toxins that block the pathogenic process [216]. The extract of guava leaf is one of its examples that exhibits a bactericidal effect and is also involved in the neutralization of toxins produced by pathogens [217]. The berberine plant alkaloid has been presented to be an effective natural product showing antibacterial activity against Staphylococcus epidermidis [218]. Nevertheless, a crude extract having multiple active compounds has fewer chances to develop AMR as compared to single isolated compounds or antibiotics [67]. Khare et al. explored different classes of phytochemicals against MDR microbial pathogens. They explained the use of plant extracts in different solvents and showed activities against MDR microbes [219].

### 8.8. Nanoparticles

**Figure 15:**
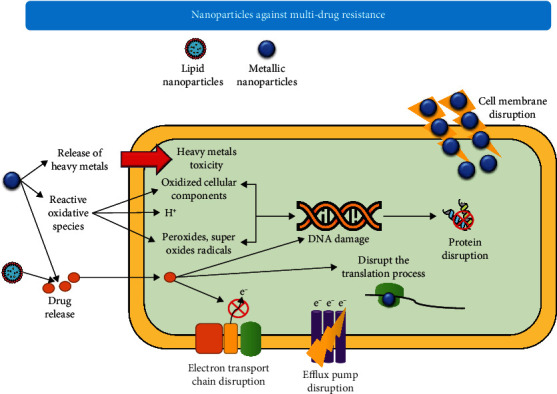
Molecular mechanisms of action of nanoparticles against multi-drug-resistant bacteria.

Nanotechnology has now been used to control antimicrobial resistance. Nanoparticles (NPs) provide a useful platform in the medical field based on their materialistic properties, i.e., high surface-to-volume ratio, supplementary attachment to a small molecule like antibiotics, and a size range equivalent to cellular systems [220]. NPs show dual actions in combating AMR. One is that they have overall bactericidal activity, and second, they act as nanocarriers for antibiotics and AMPs [221]. Functionalized monolayer-protected gold nanoparticles (AuNPs) have been proven to inhibit the growth of clinical MDR, including both Gram-positive bacteria and GNB. They interact with cell membrane AuNPs and show little toxicity against normal body cells [222]. Three basic types of nanoparticles demonstrate multiple mechanisms simultaneously to overcome antimicrobial resistance (Figure 15). The types of these NPs and their mechanisms of action are listed in Table 2. In the second case, in which NPs act as nanocarriers, antibiotics are conjugated or infused via covenant or noncovalent interaction with them [223].

Using this method, the efficiency of antibiotics is enhanced; thus, they show high efficacy at a less minimal inhibitory concentration (MIC) as compared with free antibiotics [224]. It is observed that when vancomycin and ampicillin were used in combination with AuNPs, it produced effective results at MIC against Gram-positive bacteria and GNB, respectively [225]. The presence of specific functional ligands on the surface of NPs assists in direct multivalent interactions to target biological molecules, thus permitting NPs to be used as self-therapeutic agents [226, 227].

A study reported the implementation of metallic NPs against MDR bacteria. Metallic NPs damage the membrane proteins by generating superoxide ions and free radicals that interfere with the cellular granules [228]. Majidinia et al. crucially described different nanodrug delivery systems such as metal nanoparticles, mesoporous silica, solid lipids, polymeric nanoparticles, liposomes, dendrimers, and other nanostructures against the MDR. They also explained innovative strategies such as RNA interference that interfere with membrane protein efflux functioning [229]. A study synthesized a highly stable codelivery drug microbubble triad that includes porphyrin/camptothecin-floxuridine. In vitro testing showed a substantial decrease in adenosine triphosphate- (ATP-) binding cassette subfamily G member 2 (ABCG2) expression and an increase in intracellular camptothecin combating the chemotherapy drug resistance. Further, in vivo study revealed a 90% inhibition rate of tumors in HT-29 cancer [230].

CAL02 is an advanced liposomal NP used for patient studies affected by severe pneumococcal pneumonia. CAL02 made up of sphingomyelin and cholesterol contains cell-surface specific rafts that are recognized by bacterial toxins including hemolysin and pneumolysin, phospholipase C-acting *α*-toxin, streptolysin, and tetanolysin, because of their higher binding affinity [231]. NPs are also on way to enhance the antimicrobial activity of AMPs (Figure 15). The temporin B (alpha-helical AMP) was selected to load on chitosan nanoparticles (CS-NPs), representing a long-term killing effect against *S. epidermidis*, which is a major cause of hospital infections [232]. It is experimentally investigated that the temporin B-CS-NPs produce a prolonged effect as compared to temporin B or CS-NP alone and minimize the toxic effects of the AMPs on normal cells [221]. In addition, nanocarriers are also being used for the delivery of P-gp (targeting ABC transporters) inhibitors in the case of cancer cells decreasing the MDR [233]. Latest advances in the use of hydrogels with antibiotics and nanoparticles explored the gateway to combat the MDR [234, 235]. However, it is the need of the hour to focus on in vivo and ex vivo studies to elaborate on absolute nanosystem in controlling AMR.

## 9. Future Perspectives

To trail the AMR in the future, the first and most necessary step is to raise awareness via education, cajoling, and advocacy. However, we must recognize our limitations and work to identify the reasons and mechanisms of the AMR issue. The time for traditional tactics is over. New ideas, tactics, and techniques are required to confront the situations that might challenge the circumstances having a potential for AMR emergence. It is imperative to prerequisite diagnostic technologies, automation, and economic support that will assist to overcome the AMR. We can revitalize the use of antimicrobial drugs by exploring new avenues, which may help to combat resistance.

Researchers are exploring novel ways to synthesize the new antimicrobial drugs by studying the drugs' toxicity effects on host cells and the mechanism of action involved in antimicrobial resistance. Natural compounds have shown promising antimicrobial results with cost-effectiveness. But they have some limitations regarding their isolation, characterization, and production. Therefore, it is requisite to work in their isolation and uplift to ease their use. The understanding of the structure and mechanism of action of existing AMPs will help to design new AMPs according to innovative resistance patterns. AMPs will promise a new endeavor against AMR. The combined therapy of antibiotics with phages or AMPs can probably be another good choice in the future against the blitz of AMR. It is investigated that QS inhibitors could be used to suppress the CRISPR-Cas (clustered regularly interspaced short palindromic repeats) adaptive immune system to enhance medical applications, including phage therapies and antibiotics. The CRISPR-Cas system is conserved in most classes of bacteria, so the optimization and development of new delivery systems that inhibit CRISPR-Cas may help to overcome MDR [249]. Bacteriophages, liposomes, and gene or cellular therapies offer another modern and modified strategy of administration and are presumed to be less affected by the emergence of resistant species.

There will be a need to track down new drugs with simultaneous multiple mechanisms of action to combat AMR. We should think poles apart and test long-lasting and cherished assumptions. At present, scientists are trying to make antiresistant and antibiotic nucleic acids which will assist in the upcoming future for the effective removal of ARG, thus dismantling challenging resistant strains [249]. Ground-breaking targeted delivery methods such as nanoparticles or aerosol delivery will be needed to modify in the future to increase the pharmacodynamics, PK, and bioavailability of drugs. It also recommends concentrating on tactics that have less hazardous side effects and stimulate the body's immunological response against AMR. A joint effort by biopharmaceutical companies, regulators and investors, governments, and clinical researchers is mandated to investigate viable strategies to treat infections in the future.

## 10. Conclusion

Long-term future perception of AMR is unpredictable. A look back at the beginning of antibiotic discovery helps them understand the rationale for their position. Scientists are amazed by the landmark of drug discovery and the optimistic effects in the therapeutic world, which have ultimately led to resistance developments. A French microbiologist predicted long ago that *gonococci*, *pneumococci*, and *meningococci* would not break their sensitivity to penicillin in future profiles (*Pour uneespèce qui au départétaitentièrement sensible, l'espèce sera toujoursaussi sensible. C'est le cas des germestrèssensibles à la pénicilline: gonocoques, pneumocoques, méningocoques*). But at the time had gone, we were strongly impelled toward a post-antimicrobial era in which treatment of even a common infection became tricky [250].

## Figures and Tables

**Table 1:** Types of adjuvants and their mode of action with examples.

Types of adjuvants	Functions	Approval by regulatory agencies (FDA)	Mode of action	Examples	References
Class I adjuvants					
Class I-A adjuvants	Directly suppress antibiotic resistance.	Approved	Inhibitors of serine and metallo-*β*-lactamases.	*Clavulanic acid*, *Tazobactam*, *Sulbactam*, *Avibactam*, *Relebactam*, *Vaborbactam*, *Aspergillomarasmine A*	Dortet et al. [112]; Bush [113]
Class I-B adjuvants	Enhance antibacterial activity by evading intrinsic-resistant mechanisms.	Not approved yet	Inhibitors of passive (intrinsic) resistance. Inhibitors of efflux systems.	*Loperamide*, *Murgocil*, *PAβN*	Farha et al. [114]; Li et al. [115]; Brackman et al. [116]
Class II adjuvants	Enhance antibiotic activity in the host.	Not approved yet	Immunomodulatory effect (enhance host defense mechanisms).	*Streptazolin*.	Cassone and Otvos [117]; Perry et al. [118]

FDA: Food and Drug Administration.

**Table 2:** Liposomal nanoparticles as antimicrobial agents, their mechanism of action, and target microbial agents.

Type	The main character as antimicrobial agents	Targeted microbial	Resistance development	Reference
Nitrogen oxide-releasing nanoparticles (NO-NPs)	Release RNOs → lysis of plasma membrane, inhibition of DNA repairing enzyme, and DNA deamination	MDR bacteria include *E. faecalis*, *E. coli*, *P. aeruginosa*, and MRSA	Fewer chances of resistance development due to activation of multiple antibacterial mechanisms	Blecher et al. [236]; Hajipour et al. [237]
Chitosan-containing nanoparticles (chitosan NPs)	Chitosan is a polymer and has a high positive charge density → decreases the activity of metalloprotein which is necessary for bactericidal activity	*E. coli*, *S. aureus*, and Gram-positive and Gram-negative bacteria	No resistance development because their targeted sequence is the conserved proportion of the pathogen	Blecher et al. [236]; Huh and Kwon [238]; Friedman et al. [239]
Metal-containing nanoparticles				
Silver-containing nanoparticles (Ag-NPs)	Act as bactericidal due to the Ag + ion release → create a hole in the cell membrane, inhibition blockage of ETC, and denaturation of the 30S ribosome	MDR bacteria, *P. aeruginosa*, ampicillin-resistant *E. coli*, erythromycin-resistant *S. pyogenes*, MRSA	AMR develops slowly and rarely	Iavicoli et al. [240]; Yun et al. [241]
Zinc oxide-containing nanoparticles (ZnO-NPs)	Produce Zn+, ROS, and H_2_O_2_ → damage lipid and protein of membrane and produce oxidative stress	Methicillin-resistant *Streptococcus* and MRD	Low chances of AMR development	Blecher et al. [236]; Saraf [242]
Copper-containing nanoparticles (Cu-NPs)	Strongly bind to the cell surface and produce Cu+2 and ROS → inhibition of DNA replication	*E. coli*, *S. aureus*, *Listeria monocytogenes*	Low chances of AMR development	Huh and Kwon [238]; Ahamed [243]
Titanium oxide nanoparticles (TiO-NPs)	Generate reactive ROS by photocatalysis → damage bacterial cell wall	*E. coli*, *P. aeruginosa*, *Enterococcus faecium*, *C. albicans*, and bacterial spores	Rapid develop resistance	Blecher et al. [236]; Huh and Kwon [238]
Magnesium oxide	Absorb halogen results in increased antibiotic activity → by inactivating the enzyme, damaging cell membrane, and leading to leakage of intracellular material	*E. coli*, *S. aureus*, *Bacillus*	Less chance	Blecher et al. [236]; Vidic et al. [244]
Gold nanoparticles (Au-NPs)	Have a polyvalent effect and generate ROS which produces oxidative stress and disrupts cell wall integrity	MRSA, *P. aeruginosa*	Less development of resistance	Lima et al. [245]; Lokina and Narayanan [246]
Silicon nanoparticles (Si-NPs)	Produce toxic effects which influence bacterial differentiation and spreading	*E. coli*, *S. aureus*, *C. albicans*	Easily develop	Yamamoto et al. [247]; Dhapte et al. [248]
Bismuth nanoparticles (BI-NPs)	The photoelectric effect produces free radicals and electrons	MDR, *P. aeruginosa,* and Gram-positive bacteria	Not confirmed	Blecher et al. [236]; Huh and Kwon [238]

Legends: ROS: reactive oxygen species; ETC: electron transport chain; H_2_O_2_: hydrogen peroxide.

## Data Availability

Data can be available on reasonable request.
